# Development and internal validation of a machine learning algorithm for the risk of type 2 diabetes mellitus in children with obesity

**DOI:** 10.3389/fendo.2025.1649988

**Published:** 2025-08-08

**Authors:** Jin-Xia Yang, Yue Liu, Rong Huang, Hai-ying Wu, Ya-yun Wang, Su-ying Cao, Guo-ying Wang, Jian-Min Zhang, Zi-Sheng Ai, Hui-min Zhou

**Affiliations:** ^1^ School of Medicine, Tongji University, Basic Medical Science, Shanghai, China; ^2^ Children’s Hospital of Soochow University, Endocrine Genetic Metabolism, Suzhou, China; ^3^ Gongli Hospital, Pudong New District, Department of Orthopedic Surgery, Shanghai, China; ^4^ Shanghai First Maternity and Infant Hospital, School of Medicine, Tongji University, Shanghai, China; ^5^ Children’s Hospital of Soochow University, Department of Traditional Chinese Medical, Suzhou, China

**Keywords:** obesity, children, risk prediction model, machine learning, type 2 diabetes mellitus

## Abstract

**Aim:**

We aimed to develop and internally validate a machine learning (ML)-based model for the prediction of the risk of type 2 diabetes mellitus (T2DM) in children with obesity.

**Methods:**

In total, 292 children with obesity and T2DM were enrolled between July 2023 and February 2024 and followed for at least 1 year. Eight ML algorithms (Decision Tree, Logistic Regression, Support Vector Machine (SVM), Multilayer Perceptron, Adaptive Boosting, Random Forest, Gradient Boosting Decision Tree, and Extreme Gradient Boosting) were compared for their capacity to identify key clinical and laboratory characteristics of T2DM in children and to create a risk prediction model.

**Results:**

Forty-nine children were diagnosed with T2DM during the follow-up period. The SVM algorithm was the best predictor of T2DM, with the largest area under the receiver operating characteristic curve (0.98) and accuracy (93.2%). The SVM algorithm identified eight predictors: BMI, creatinine, prealbumin, glucose (180 min), glycosylated hemoglobin A1c, thyrotropin, total thyroxine (T4), and free T4 concentrations. Thus, an ML-based prediction model accurately identifies children with obesity at high risk of T2DM. If externally validated, this tool could facilitate early, personalized interventions aimed at preventing T2DM.

**Discussion:**

The rising prevalence of obesity in childhood is associated with an increase in the risk of early-onset T2DM. Therefore, the early identification of individuals at high risk is crucial to prevent the development of this disease. In a comparative analysis of the performance of multiple ML algorithms, we found that the SVM algorithm was the best predictor of the development of T2DM.

## Introduction

Obesity is a chronic metabolic disease that is characterized by excessive fat deposition, and it is one of the top 10 chronic diseases worldwide, according to the World Health Organization (WHO) (1). In recent years, the number of children with obesity has been increasing, especially in Asia (1). According to the WHO, the global number of children <5 years of age with obesity or overweight reached 41 million in 2016, and children in Asia accounted for 48% of the total (2). The latest edition of the World Obesity Report, published on March 3, 2024 by the World Obesity Federation, states that by 2035, the number of children and adolescents 5–19 years old with overweight or obesity worldwide will rise to 770 million. Furthermore, between 2020 and 2035, an annual growth rate of 2.0% in childhood overweight or obesity is predicted, such that the prevalence of overweight or obesity in this age group will be 72% in 2035. Thus, obesity has become a serious public health problem that jeopardizes children’s health worldwide (3, 4).

Childhood obesity is an important risk factor for type 2 diabetes mellitus (T2DM). Furthermore, with the increasing global prevalence of obesity, the number of children and adolescents with T2DM has been rising year on year. An epidemiologic dataset collected in the United States showed that the prevalence of T2DM in children and adolescents increased from 34/100,000 in 2001 to 46/100,000 in 2009, and 67/100,000 in 2017 (5). Large longitudinal cohort studies have shown that the incidence and prevalence of T2DM and comorbidities are increasing rapidly, and this is seriously affecting the physical and mental health of children and adolescents and increasing the burdens associated with their prevention and control. The trend in the prevalence of T2DM in children and adolescents in China has paralleled the trend in the prevalence of obesity in this group. Specifically, the prevalence of T2DM increased in China from 4.1/100,000 in 1995 to 10.0/100,000 in 2010 (6, 7).

The prevalence of diabetes in children is of global concern, and in particular the prevalence of T2DM is increasing in children and adolescents, secondary to an increase in the prevalence of obesity in childhood (8, 9). However, it may not be cost effective to screen for T2DM in the general population or in every young person with overweight or obesity. The U.S. Preventive Services Task Force concluded that the available evidence is insufficient to assess the benefits and drawbacks of T2DM screening in children and adolescents (10). In addition, the International Society for Pediatric and Adolescent Diabetes (ISPAD) guidelines recommend that only children and adolescents with significant risk factors for diabetes are screened (11). Although the prevalence of T2DM in children and adolescents is increasing, universal screening of adolescents is not currently recommended owing to the high cost. However, the early screening of individuals with risk factors can increase treatment success, improve quality of life, and delay the development of diabetes-related complications. Predictive models could help with the early identification of children and adolescents with overweight or obesity who are at risk of diabetes, and the identification of specific predictors should inform the subsequent development of risk prediction tools.

Recent epidemiological studies indicate that the incidence of T2DM in children with obesity is increasing at an annual rate of 4%–5%, with these individuals facing a 4- to 7-fold higher risk than that of their normal-weight peers (12). Early-onset T2DM is particularly concerning owing to its aggressive disease progression, with accelerated β-cell deterioration and earlier onset of diabetic complications compared with those for adult-onset cases. Although guidelines from the American Diabetes Association (ADA) recommend annual HbA1c screening for obese children, current risk stratification tools—such as BMI percentile cutoffs and oral glucose tolerance tests (OGTT)—exhibit suboptimal sensitivity (52–68%) and specificity (71–79%), leading to significant underdiagnosis (13).

Conventional risk prediction models for T2DM, predominantly based on logistic regression, fail to capture complex, nonlinear interactions among metabolic, genetic, and lifestyle factors (14). Machine learning (ML) has emergedas a promising approach to address these gaps. ML models can potentially analyze large-scale population based datasets, incorporating a wide range of variables to predict the probability of developing T2DM and its associated complications and mortality (15). While ML algorithms have demonstrated superior predictive performance for T2DM in adults, their application in pediatric populations remains underexplored. Existing pediatric models often rely on static clinical variables, neglecting dynamic growth patterns, longitudinal metabolic changes, and socioenvironmental determinants—critical factors influencing diabetes risk in children (16). Furthermore, most algorithms lack interpretability, limiting their clinical utility for decision-making in pediatric endocrinology.

To address these gaps, we developed and internally validated an interpretable ML algorithm to predict the risk of T2DM in children with obesity. Our study introduces several innovations, including a multidimensional feature matrix integrating dynamic growth trajectories, metabolic biomarkers, and socioenvironmental exposures. We evaluated eight ML algorithms (Decision Tree, Logistic Regression, Support Vector Machine (SVM), Multilayer Perceptron, Adaptive Boosting, Random Forest, Gradient Boosting Decision Tree, and Extreme Gradient Boosting) for the capacity to identify the key clinical and laboratory characteristics of T2DM in children. By leveraging high-dimensional data and robust validation, this study provides a basis for early, personalized interventions for high-risk pediatric populations, ultimately mitigating the long-term burden of T2DM.

## Materials and methods

### Study sample and design

The study was reviewed by the hospital ethics committee, the final approval number of ethics was 2024CS141. Ethical review content includes scientific approval of research projects, review of observational study protocols, human genetic resource management commitment letters, explanations of clinical research project funding sources or statements of no self-funded support, clinical research agreements, etc. In this study, all the children’s parents agreed to participate and signed the informed consent form, and all the children’s informed consent was signed by the parents together in the same informed consent form.

Between July 2023 and February 2024, children with obesity who attended outpatient clinics or were admitted to wards of the Department of Endocrinology, Genetics, and Metabolism at the Children’s Hospital of Soochow University were selected using simple random sampling. From patients with pediatric obesity, we randomly selected participants using a random number sequence to ensure representativeness. To ensure reproducibility, all random processes were controlled using Python numpy pseudorandom number generator with a fixed seed.

The inclusion criteria for the obesity/no T2DM group were age <18 years old and the presence of obesity. According to the simple diagnostic criteria for obesity in “Zhufutang Practical Pediatrics” (8th edition), an actual body mass of >20% based on the standard height and body mass of the reference population is defined as obesity. The formula for calculating the height-to-weight ratio (%) is as follows: Standard weight (kg) for children of the same height/Actual weight (kg) * 100. The height-to-weight ratios for mild, moderate, and severe obesity were defined as 20.1% to 30.0%, 30.1% to 50.0%, and ≥50.1%, respectively. Informed consent from the child’s father or mother. The exclusion criteria for this group were the presence of other serious chronic systemic diseases (such as congenital heart disease, epilepsy, and blood disorders), the presence of secondary obesity caused by a specific disease or drug, and the use of lipid-lowering drugs.

The inclusion criteria for the obesity/T2DM group were age <18 years old, and a diagnosis of obesity, categorized as described above, and a diagnosis of T2DM. Diabetes mellitus was diagnosed in children using a two-step process. The first step was to identify the presence of diabetes mellitus of any type, using the criteria of the American Diabetes Association (ADA) or ISPAD (the criteria of the ADA are for the definition of diabetes mellitus in adults). Diabetes mellitus was diagnosed when symptoms of hyperglycemia (polyphagia, polydipsia, polyuria, and/or unexplained weight loss) occur alongside one of the following laboratory findings: blood glucose concentration (FPG) ≥7.0 mmol/L; blood glucose concentration 2 h after a glucose load ≥11.1 mmol/L; HbA1c ≥6.5%; or a random glucose concentration ≥ 11.1 mmol/L. If there were no clear symptoms of hyperglycemia, any abnormal laboratory findings were confirmed by retesting on a subsequent occasion. The second step was to determine the type of diabetes present. Glutamic acid decarboxylase antibody, islet cell antigen 2 antibody, zinc transporter 8 antibody, and insulin autoantibody testing were performed because islet autoimmunity is common in children who have been clinically diagnosed with T2DM. Genetic testing should be performed when necessary to confirm mature-onset diabetes of the young. Informed consent from the child’s father or mother. The exclusion criteria for this group were the presence of secondary obesity; the presence of an acute complication of diabetes mellitus, such as ketoacidosis; the presence of another serious chronic systemic disease, such as heart disease or kidney disease; the use of a lipid-lowering drug; and the recent use of a drug that might affect the blood glucose concentration.

Data collection and quality control methods: Prior to data collection, the researchers designed a data collection form that included the following content: General clinical data, Laboratory test data, Diabetes-related indices. All survey information was obtained from the patients’ case records and filled in. This study strictly adhered to the inclusion and exclusion criteria for research subjects. Researchers who had undergone standardized training used a standardized script to provide survey participants with detailed explanations of the purpose and significance of the study, as well as the parts of the research process that required the cooperation of the participants. Prior to entering the study, written informed consent was obtained from the parents of the children to ensure their informed consent. During the study, a total of 300 cases were included. After excluding invalid data such as missing survey items, 292 cases were ultimately included.

### Laboratory measurements and clinical characteristics of the participants

1. General clinical data: the sex, ethnicity, year of birth, growth and development data, family history, history of drug allergy, history of previous illnesses, duration of the disease, clinical symptoms, use of medication, history of control of blood glucose or HbA1c, height, body mass, and body mass index (BMI; body mass (kg)/[height (m)]^2^ of the participants were collected.
2. Laboratory test data: routine biochemical data, indices of glucose and lipid metabolism, glucocorticoid-related indices, diabetes autoantibody titers, indices of thyroid function, and urine protein profile data were collected.
3. Diabetes-related indices: the homeostasis models of assessment-insulin resistance (HOMA-IR) and β-cell function (HOMA-β) were calculated as follows.

HOMA-IR = fasting blood glucose (mmol/L) × fasting insulin (mIU/L)/22.5. The values obtained were converted to grades as follows: grade I (mild insulin resistance): HOMA-IR <3.0; grade II (moderate insulin resistance): HOMA-IR: 3.0–5.0; and grade III (severe insulin resistance): HOMA-IR ≥5.0.

HOMA-β = fasting insulin (mIU/L)/[fasting blood glucose (mmol/L) × 3.5]. A normal HOMA-β value is 100%; a low HOMA-β value suggests that pancreatic islet β-cell function is poor and a high value suggests substantial pancreatic islet β-cell secretion. Grading criteria were as follows: greater than 50% indicates normal pancreatic β-cell function or good compensatory ability, less than 50% indicates impaired pancreatic β-cell function, and greater than 300% may indicate early compensatory stage of diabetes.

### Statistical analysis

Statistical analysis was performed using SPSS v.24.0 software (IBM, Inc., Armonk, NY, USA) and Python software v.3.10.4 released by the Python Software Foundation on March 24, 2022. Normally distributed clinical data are described using mean ± standard deviation; Student’s *t*-tests were used for comparisons between two groups, and one-way ANOVA plus Tukey’s *post-hoc* tests were used for comparisons among multiple groups. Non-normally distributed clinical data are described using median (first quartile, third quartile); two groups were compared using the Wilcoxon rank-sum test, and multiple groups were compared using the Kruskal–Wallis test, with *P*-values adjusted using the Benjamini–Hochberg correction. Categorical data are expressed as numbers (percentages) and were analyzed using the chi-square test and ANOVA was used with *post hoc* tests. Logistic regression models were used to compare the T2DM and No T2DM groups with respect to clinical, demographic, and laboratory data diabetes-related indices. *P* < 0.05 was regarded as indicating statistical significance.

### Model development

Before developing the machine learning algorithm, we compared the differences in patient data between “obese patients without T2DM” and “obese patients with T2DM,” We then further incorporated data with statistically significant differences (P<0.05) into a logistic regression model. Ultimately, we determined the predictors through the logistic regression model and developed and evaluated machine learning models for data with statistical differences. Eight machine learning algorithms were used to select the key parameters that differentiated the groups and build risk prediction models: Decision Tree (DT), Logistic Regression (LR), Support Vector Machine (SVM), Multilayer Perceptron (MLP), Adaptive Boosting (AdaBoost), Random Forest (RF), Gradient Boosting Decision Tree (GBDT), and Extreme Gradient Boosting (XGBoost), (**Table 1**). Using simple random sampling, the participants were randomly allocated to training (75%) and validation (25%) sets, and the models were developed using the training set (75% of data) and internally validated using the validation set (25% of data). Ten-fold cross-validation was performed using the training set, with one-tenth of these data being reserved for testing and each of the remaining nine-tenths being used in turn for training. Nomograms were constructed using the results of the ML models. To control overfitting or underfitting issues in machine learning models, we implemented measures such as data preprocessing and partitioning, cross-validation, and model selection. First, the code performed thorough data preprocessing, including missing value imputation, standardization, and one-hot encoding. Continuous features are imputed using the median of the training set, while categorical features are imputed using the mode (or 0 if all values are missing). Categorical variables are converted to integer type. This approach avoids model instability caused by missing data or different scales, thereby reducing the risk of underfitting. Subsequently, continuous variables are standardized to ensure that the mean of the features is 0 and the variance is 1. This is particularly important for algorithms such as logistic regression, SVM, and neural networks to prevent model bias caused by certain features being too large in scale. Additionally, categorical variables are one-hot encoded to introduce them into the model (one-hot encoding increases the feature dimension, and during training, align ensures that missing dummy variable columns in the test set are filled with 0). Second, during model training, a cross-validation strategy was introduced to effectively mitigate overfitting risks. The best parameters were selected based on the average score from cross-validation, thereby avoiding overfitting configurations caused by the randomness of a single split. In addition to the above-mentioned hyperparameter and structural control for each model, the code also focused on metrics such as ROC AUC in model evaluation. Overall, these eight models achieved similar performance on the training and validation sets through various measures such as data cleaning, regularization hyperparameters, and cross-validation selection, without showing obvious overfitting (such as validation scores significantly higher than test scores) or underfitting (such as low training and validation scores). The SMOTE oversampling method was used to address the imbalance in the training data during model development and evaluation. In addition, SMOTE was applied to the training set prior to grid search training of each model, synthesizing oversampling of the minority class to balance the class distribution. Currently, no category weights are used in model training to reduce majority class bias.

**Table 1:** Hyperparameters of machine learning algorithms.

Model	Param grid range	Best params
DT	max_depth: [5, 10, 20]; min_samples_split: [2, 5, 10]	max_depth=5; min_samples_split=5
LR	C: [0.1, 1, 10]; solver: [‘liblinear’, ‘saga’]	C=0.1; solver=‘liblinear’
SVM	C: [0.1, 1, 10]; kernel: [‘linear’, ‘rbf’]	C=10; kernel=‘rbf’
MLP	hidden_layer_sizes: [(50), (100), (150)],; activation: [‘relu’, ‘tanh’]	hidden_layer_sizes=(50),; activation=‘relu’
AdaBoost	n_estimators=50, learning_rate=1.0	No tuning (defaults)
RF	n_estimators: [50, 100, 200]; max_depth: [10, 20, 30]	n_estimators=50; max_depth=10
GBDT	n_estimators=100, learning_rate=0.1, max_depth=3	o tuning (defaults)
XGBoost	n_estimators: [50, 100, 200]; learning_rate: [0.01, 0.1, 0.3]	n_estimators=50; learning_rate=0.3

### Assessment of model performance

Five key metrics were used to evaluate the efficacy of the models: Cross-Validation (CV) accuracy, Area Under the Curve (AUC), overall accuracy (ACC), recall, and F1 score. AUC values between 0.5 and 0.7 indicated a low level of accuracy, values between 0.7 and 0.9 indicated a moderate level of accuracy, and values >0.9 indicated a high level of accuracy.

## Results

### Basic characteristics of the participants

Forty-nine patients developed T2DM (16.8%) during the follow-up period. The mean age of the participants was 11.96 ± 2.29 years, and 162 (55.5% of 292 total participants) were male. The laboratory test data and demographic characteristics of the participants are shown in **Table 2**.

**Table 2:** Comparison of groups with and without type 2 diabetes mellitus (T2DM).

Item	No T2DM (n=243)	T2DM (n=49)	Statistical value	P value*
Age (year)	11.79 ± 2.26	12.80 ± 2.25	8.145	0.005
Weight	72.33 ± 18.05	64.07 ± 18.04	8.555	0.004
Height	157.42 ± 12.34	162.41 ± 11.17	6.883	0.009
BMI	28.77 ± 4.15	24.03 ± 5.48	45.033	<.001
Lipase	25.51 ± 5.31	33.85 ± 50.86	6.271	0.013
Total Bilirubin	11.00 ± 4.91	13.62 ± 5.25	11.334	<.001
Direct Bilirubin	3.74 ± 1.66	4.41 ± 1.55	6.884	0.009
Aspartate aminotransferase	31.46 ± 18.19	38.76 ± 42.41	3.785	0.053
Creatine kinase	115.27 ± 52.03	99.39 ± 62.16	3.549	0.061
Lactate dehydrogenase	252.56 ± 61.96	224.52 ± 58.70	8.496	0.004
Lactate dehydrogenase	188.67 ± 48.82	165.32 ± 39.46	9.896	0.002
Glycocholic Acid Measurement	1.52 ± 0.68	2.41 ± 5.20	6.642	0.01
Glucose	5.09 ± 0.64	7.78 ± 4.32	85.549	<.001
Glucose 0 min	4.50 ± 0.58	6.03 ± 1.59	135.677	<.001
Glucose 30 min	7.95 ± 1.38	8.72 ± 2.22	10.1	0.002
Glucose 60 min	8.03 ± 1.88	11.54 ± 3.40	103.338	<.001
Glucose 120 minutes	6.94 ± 1.61	12.19 ± 3.97	236.303	<.001
Glucose 180 minutes	5.38 ± 1.45	10.64 ± 3.83	270.06	<.001
Glycated hemoglobin A1c	5.55 ± 0.47	10.64 ± 3.83	297.242	<.001
Insulin Measurement	612.18 ± 348.82	236.72 ± 242.80	51.65	<.001
Serum C-peptide measurement	4.84 ± 1.72	1.80 ± 1.40	135.587	<.001
LDL Cholesterol	2.66 ± 0.61	3.00 ± 1.00	10.112	0.002
Triglycerides	1.29 ± 0.68	1.64 ± 1.57	6.263	0.013
Total Cholesterol	4.39 ± 0.75	4.79 ± 1.19	9.612	0.002
Apolipoprotein B	0.80 ± 0.17	0.91 ± 0.28	13.89	<.001
Cortisol Measurement (AM)	405.83 ± 183.93	472.08 ± 189.88	5.232	0.023
Thyrotropin	2.90 ± 1.48	2.35 ± 0.91	6.434	0.012
Total T3	1.98 ± 0.17	1.80 ± 0.39	25.407	<.001
Free T3	6.16 ± 0.69	5.75 ± 0.84	13.865	<.001
Free T4	15.30 ± 1.77	17.06 ± 2.59	33.764	<.001
Urine Creatinine	10513.93 ± 3380.19	9300.64 ± 5313.10	4.225	0.041
Urine Immunoglobulin G	4.45 ± 5.51	9.20 ± 25.47	6.941	0.009
Urinary Microalbumin (UMAL)	20.98 ± 45.35	46.09 ± 136.34	5.365	0.021
Porphyrins, Porphyrites, Porphyrites	3.49 ± 5.89	8.14 ± 29.10	5.207	0.023
Alpha 1 Microglobulin	10.39 ± 4.37	12.09 ± 8.16	4.369	0.037
Cortisol	0.44 ± 0.22	0.62 ± 0.32	21.23	<.001
Glycosaminoglycosidase	10.62 ± 3.59	12.15 ± 8.81	4.034	0.046
Insulin Resistance Index HOMA-IR	20.35 ± 11.57	10.92 ± 12.65	26.246	<.001
Pancreatic β-cell secretion index	3.82 ± 0.49	4.26 ± 0.68	60.899	<.001

*ANOVA.

### Laboratory data and clinical characteristics of the participants

There were significant differences (*P*<0.05) between the T2DM group and the No T2DM group with respect to the age, body mass; height; BMI; lipase activity; total or direct bilirubin concentrations; aspartate aminotransferase, creatine kinase, lactate dehydrogenase, or alpha hydroxybutyrate dehydrogenase activities; glycocholic acid, random glucose, glucose 0, 30, 60, 120, or 180 min post-glucose load concentrations; glycosylated hemoglobin A1c level; insulin, C-peptide, Low-Density Lipoprotein (LDL)-cholesterol, triglyceride, total cholesterol, apolipoprotein B, cortisol, thyrotropin, total T3, free T3, or free T4 concentrations; urinary creatinine, immunoglobulin G, microalbumin, or transferrin concentrations; α1-microglobulin, β2-microglobulin, or *N*-acetylaminoglucosidase concentrations; HOMA-IR; or HOMA-β.

### Key predictors

Variables included in the logistic regression analysis included BMI, creatinine, prealbumin, glucose (180 min), glycosylated hemoglobin A1c, thyrotropin, total T4, and free T4, as shown in **Table 3**.

**Table 3:** Logistic regression analysis of factors associated with T2DM.

Items	B	Standard error	Wald degrees of freedom significance	Significance	Exp(B)
BMI	.676	.207	10.691	.001	1.967
Creatinine	-.153	.067	5.189	.023	.858
Pre-Albumin	-.037	.019	3.623	.057	.964
Glucose 180 min	-1.351	.369	13.368	<.001	.259
Glycated hemoglobin A1c	-3.995	1.481	7.275	.007	.018
Thyrotropin	.946	.470	4.048	.044	2.576
Total T4	-.116	.048	5.771	.016	.891
Free T4	-.663	.388	2.917	.088	.515
constant	56.174	16.571	11.491	<.001	2488298875938576000000000.000

R^2^ = 0.544.

### Development of machine learning algorithms

**Figure 1:**
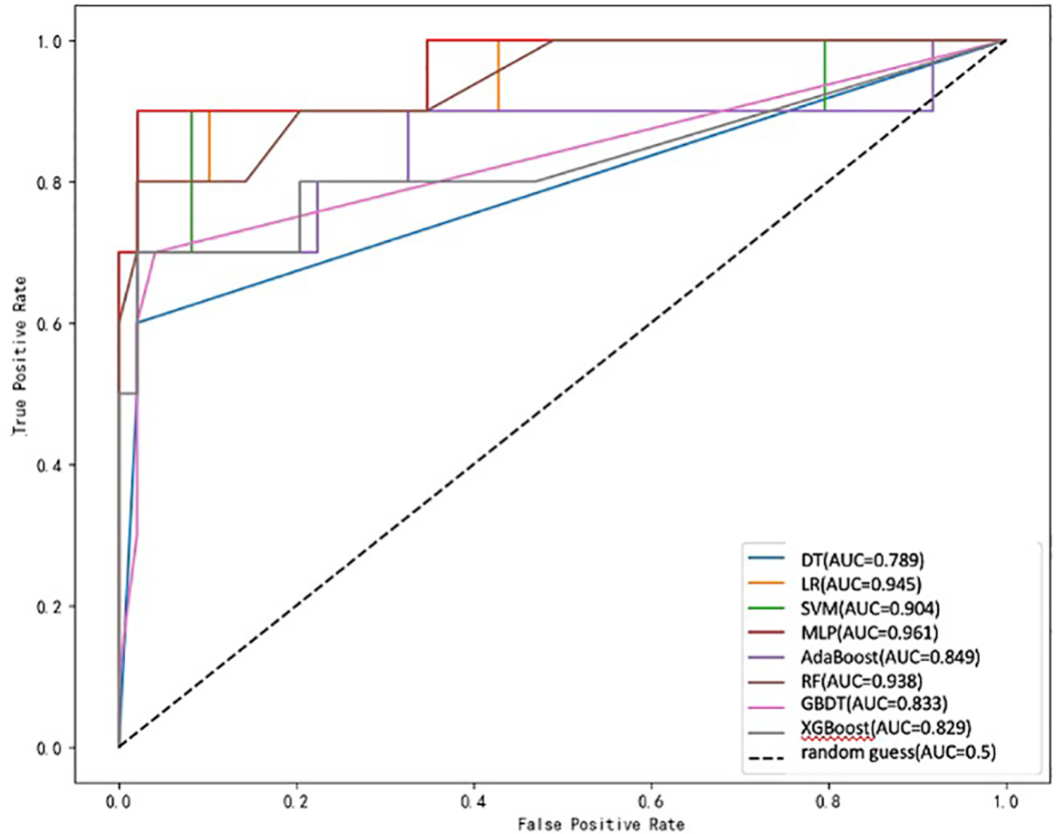
ROC curve for each prediction model.

**Figure 2:**
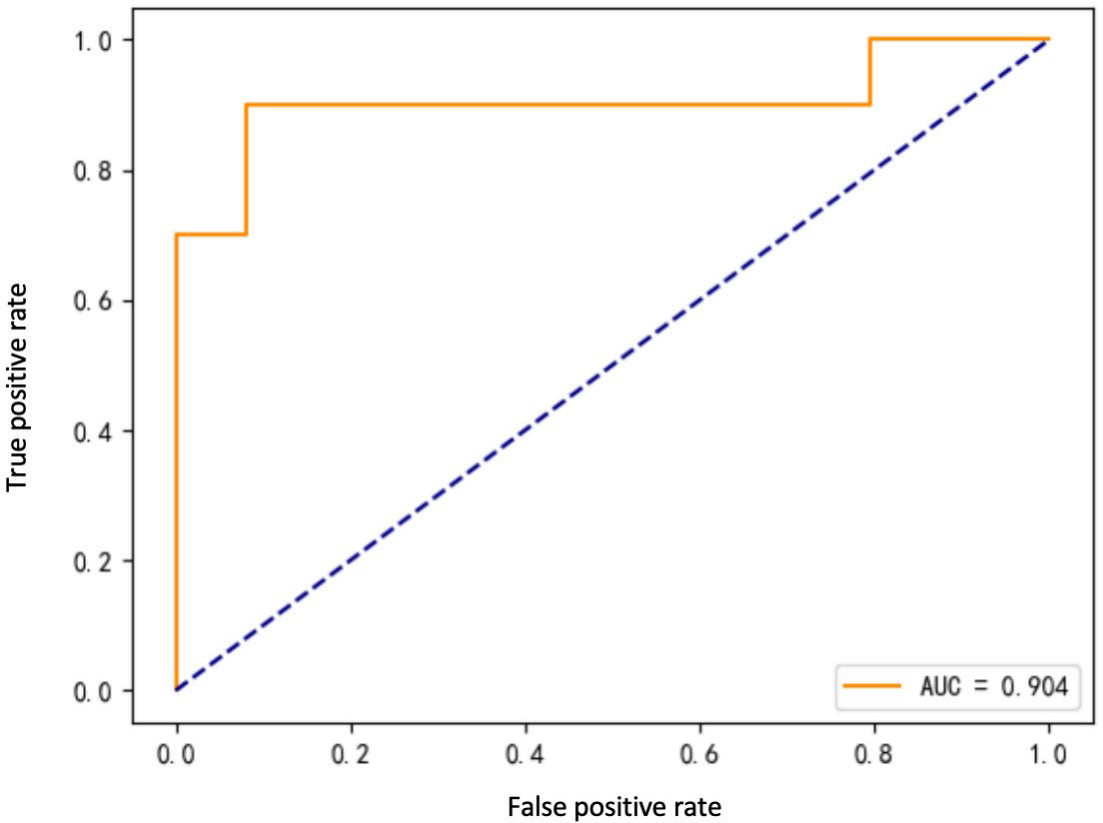
ROC curve for SVM model.

Eight ML algorithms were used (DT, LR, SVM, MLP, AdaBoost, RF, GBDT, and XGBoost) to identify the key clinical and pathologic characteristics of the T2DM group and to create a risk prediction model for T2DM in children. The SVM algorithm was the best predictor of the progression to T2DM because it showed the highest AUC (0.98) and ACC (93.2%) (**Table 4**, **Figure 1**). The eight models have distinct characteristics. DT is intuitive and interpretable and does not require feature scaling; however, it is prone to overfitting. LR outputs calibrated probabilities and has strong clinical interpretability but only captures linear relationships. SVM is effective for classification in high-dimensional spaces but is sensitive to hyperparameters and has high computational costs (**Figure 2**). MLP models complex nonlinear relationships but requires a large amount of data and has black-box characteristics. AdaBoost can iteratively correct error samples and is sensitive to noisy data. RF is resistant to overfitting and assesses feature importance; however, it may underestimate the impact of extreme values. GBDT has high predictive accuracy and handles mixed data types but has long training times. XGBoost prevents overfitting through regularization and supports parallel computing; however, hyperparameter tuning is complex.

**Table 4:** Results of a validation study of the machine learning algorithms.

Model	CV accuracy	AUC	Accuracy	Recall	F1 score
DT	0.961	0.789	0.8983	0.5000	0.6250
LR	0.974	0.945	0.9153	0.5000	0.6667
SVM	0.983	0.904	0.9322	0.6000	0.7500
MLP	0.978	0.961	0.9153	0.5000	0.6667
AdaBoost	0.970	0.849	0.8983	0.5000	0.6250
RF	0.974	0.938	0.9153	0.5000	0.6667
GBDT	0.957	0.833	0.9153	0.5000	0.6250
XGBoost	0.983	0.829	0.8983	0.5000	0.6250

The ROC curve evaluates the performance of the classifier by plotting the true positive rate against the false-positive rate at different thresholds. As shown in **Figure 1**, the MLP model yielded the highest AUC value of 0.961, indicating that it was the best classifier of the test data. The LR and RF models also yielded high AUC values of 0.945 and 0.938, respectively, and the DT model yielded the lowest AUC value of 0.789, representing a relatively poor performance. The other models (SVM, AdaBoost, GBDT, and XGBoost) yielded AUCs between 0.8 and 0.9, indicative of moderate performance. However, **Table 4** shows that SVM performed best with respect to a combination of the AUC and F1 score, with values of 0.904 and 0.7500, respectively. MLP and XGBoost performed best with respect to CV accuracy, both yielding values of 0.983. SVM performed best with respect to accuracy, yielding a value of 0.9322. The recall value was 0.5000 for all the models except for SVM, which yielded a value of 0.6000. The F1 score was highest for SVM (i.e., 0.7500). Thus, overall, SVM performed best using the present set of data, especially with respect to the AUC and F1 score.

## Discussion

### Pathogenesis of type 2 diabetes mellitus

Obesity is closely associated with the development of T2DM and the two are linked at multiple levels, with insulin resistance being a key defect. Many previous studies have shown that obesity increases the risk of insulin resistance and T2DM, but not all individuals with obesity develop T2DM, and some maintain their insulin sensitivity and relatively normal metabolism (12–16). Numerous studies have shown associations of non-esterified fatty acids, metabolites, and pro-inflammatory molecules with obesity, insulin resistance, and T2DM, and some of these can be used as specific markers for the presence of a combination of obesity and T2DM (17–23).

### Predictors of the development of diabetes in obese children

The predictors identified in this study reflect both the pathophysiological mechanisms of diabetes and the metabolic characteristics of obese children and have important clinical significance and statistical value. (1) With respect to the predictive value of glucose metabolism-related indicators, glucose (180 minutes) and glycated hemoglobin A1c, which were included in the model, are core indicators for assessing glucose metabolism status. Glucose (180 minutes) serves as a critical time point in the oral glucose tolerance test, sensitively reflecting the degree of insulin resistance and beta-cell function in children. Glycated hemoglobin A1c, as an indicator of long-term blood glucose control, also demonstrated good predictive efficacy in this model. Notably, in obese children, even when A1c is within the normal high range (5.7%–6.4%), the risk of diabetes is significantly increased. This supports the prospective value of the American Diabetes Association’s use of A1c ≥ 5.7% as a screening criterion for high-risk populations. (2) One of the innovative findings of this study is the important role of thyroid function indicators (thyroid-stimulating hormone, total T4, and free T4) in diabetes prediction. Research data shows that the higher the T4 level, the lower the risk of developing type 2 diabetes mellitus (B=-0.116, P=.016), indicating that high T4 levels are a protective factor against the development of type 2 diabetes mellitus. Thyroid hormones may influence glucose metabolism through multiple mechanisms, including regulating basal metabolic rate and energy expenditure, affecting insulin sensitivity and glucose transport, and regulating hepatic gluconeogenesis and glycogenolysis. Our data show that TSH levels are correlated with diabetes risk, suggesting that a comprehensive assessment of thyroid function is indispensable in evaluating diabetes risk in obese children. A study conducted in Anhui, China, in 2025 showed that changes in thyroid function parameters (such as elevated TSH, decreased FT3, or decreased FT4) are associated with increased inflammatory activity and impaired glucose and lipid metabolism in patients with type 2 diabetes mellitus (T2DM) (24). Additionally, TSH levels are associated with an increased risk of diabetic microvascular complications, such as nonproliferative diabetic retinopathy. This also suggests that changes in thyroid hormone levels (such as TSH and FT4) may serve as potential predictive indicators for diabetes and its complications (such as diabetic nephropathy and retinopathy). This finding is consistent with the innovative discoveries of this study. (3) With respect to the predictive significance of renal function and nutritional status markers, the inclusion of creatinine and prealbumin reflects the model’s comprehensive consideration of multi-system effects. Elevated creatinine levels may indicate early renal dysfunction, and renal insufficiency may further exacerbate glucose metabolism disorders. Prealbumin, as a sensitive indicator of nutritional status and protein-energy metabolism, often predicts metabolic reserve depletion when levels are reduced. Children with obesity often have micronutrient deficiencies and protein metabolism abnormalities, making prealbumin an important indicator for predicting metabolic outcomes.

### Clinical implications of the modeling

The early identification of children who are at high risk for obesity is critical to facilitate preventive interventions, but the existing tools, such as the ADA guidelines, comprise single threshold values. The strength of the optimal ML model identified in the present study is its compatibility with personalized risk stratification. Unlike with the population-based thresholds, ML models provide personalized risk assessments that enable clinicians to prioritize high-risk individuals. In addition, in the present study, we have comprehensively evaluated potential predictors, which included anthropometric, laboratory, and metabolic data and indices. Using these we developed and internally validated an ML algorithm for the prediction of the risk of T2DM in children with obesity. We aimed to evaluate the performance of a range of ML models for the prediction of the risk of T2DM and to select the most suitable model for use in the clinic. The selected model identified 180-min glucose, HbA1c, and BMI as the best predictors, consistent with the established screening guidelines for pediatric diabetes (6). A SHAP value analysis of data from three independent global cohorts showed that age is the most important predictor of T2DM, followed by fasting blood glucose, hemoglobin, γ-glutamyltransferase levels, and body mass index (15, 25–28). However, unlike conventional risk scores, ML can capture nonlinear relationships (e.g., relationships with creatinine and prealbumin concentrations), which may account for its greater accuracy than those of previous models.

### Comparison of model performance

As shown in **Table 4**, SVM performed well according to several assessment metrics. Specifically, SVM achieved high scores for F1 score (0.7500), indicating that it performs best with respect to the balancing of true and false-positive rates. In addition, the accuracies of SVM (0.9322) and AUC (0.904) were relatively high, second only to MLP and XGBoost. These results are consistent with those of previous studies, showing that SVM performs well with unbalanced datasets (29).

MLP and XGBoost performed the best with respect to CV accuracy, with both achieving values of 0.983, implying that they have high levels of stability and accuracy for cross-validation. However, despite the excellent performance of these two models in terms of CV accuracy, they had relatively low AUC values of 0.961 and 0.829, respectively, which may indicate that they are less appropriate for use with unbalanced datasets. This finding is consistent with those of Hastie et al. (30), who showed that the CV accuracy of a model may not fully reflect its performance with unbalanced datasets. LR and RF also performed well with respect to CV accuracy, yielding values of 0.974 and 0.974, respectively, but the values for AUC were slightly lower than those for SVM and MLP. Nevertheless, RF outperformed most other models with respect to AUC (0.938), implying that it is particularly useful for the analysis of complex data. This finding is consistent with those of Breiman et al. (31), who demonstrated the utility of RF for the analysis of high-dimensional data. AdaBoost and GBDT showed more balanced performance with respect to all the evaluation metrics but performed slightly worse than SVM and RF with respect to AUC and F1 score. This may be related to a lack of suitability of these models for the analysis of complex data. The study by Freund and Schapire (32) also showed that AdaBoost may require further optimization to improve performance.

It is worth noting that the recall value of 0.5000 that was achieved for all the models except SVM suggests that these models have some shortcomings in the identification of positive class samples, i.e., for the prediction of a high risk of T2DM. The recall of 0.6000 for SVM is substantially higher than that for the other models, which may indicate its superiority with respect to unbalanced datasets. This result is consistent with that of Joachims et al. (33), who demonstrated the superiority of SVM with respect to unbalanced data.

SVM also performed best with respect to F1 score (0.7500), implying that it has a good balance between precision and recall. MLP and RF also yielded relatively high F1 scores (0.6667), suggesting that they have some advantages for the analysis of specific types of data. This is consistent with previous findings that suggest that these models may be more effective for certain applications (34).

In addition, we also outlining plans for multi-center validation now that internal performance is established and future external validation performed using different populations in multiple centers is needed to ensure the wide applicability of the model.

### Considerations involved in model selection

When selecting the best model, multiple evaluation metrics should be used. Although SVM performed well with respect to several metrics, it performed slightly less well than MLP and XGBoost in terms of CV accuracy. Therefore, for practical applications involving complex and unbalanced data, SVM may be preferable, whereas if model stability and accuracy are the primary considerations, MLP and XGBoost may be more appropriate. In addition, RF performs well with complex data structures, but it was slightly inferior to SVM with respect to AUC and F1 score. Therefore, RF may need to be further optimized for use in scenarios in which precision and recall need to be balanced. This observation is consistent with the results of previous studies, which emphasize the need to consider multiple factors when selecting the most appropriate model (35).

### Limitations

The present study was a retrospective single-center study, and unmeasured confounders (e.g., genetic predisposition) may have affected the predictions made. Therefore, future external validation performed using different populations in multiple centers is needed to ensure the wide applicability of the model. Furthermore, the inclusion of longitudinal changes, such as in body mass, might also improve the predictive accuracy of the model. In addition, the average age was significantly younger in the obese children group than in the diabetic children group, suggesting that age is an important confounding variable. This also suggests that obese children may experience a gradual increase in insulin resistance as they age and that β-cell dysfunction typically manifests around puberty, which may explain the older average age in the diabetic group.

## Conclusion

In this study, we compared the performance of multiple ML algorithms for the prediction of the risk of T2DM in children with obesity. We found that SVM performs well with respect to several evaluation metrics, especially with unbalanced datasets. MLP and XGBoost performed the best with respect to CV accuracy but were slightly inferior to SVM with respect to AUC and F1 score. RF performed well with complex data but was slightly inferior to SVM with respect to the balance between precision and recall. Future studies should further evaluate the performance of these models with different datasets and applications to determine the optimal model selection strategy. In addition, the ease of interpretation and flexibility of the model are also important to consider when selecting a model for use in the clinic.

## Data Availability

The original contributions presented in the study are included in the article/supplementary material, further inquiries can be directed to the corresponding authors.
